# The Impact of Education and Age on Metabolic Disorders

**DOI:** 10.3389/fpubh.2020.00180

**Published:** 2020-05-20

**Authors:** Christopher R. Stephens, Jonathan F. Easton, Adriana Robles-Cabrera, Ruben Fossion, Lizbeth de la Cruz, Ricardo Martínez-Tapia, Antonio Barajas-Martínez, Alejandro Hernández-Chávez, Juan Antonio López-Rivera, Ana Leonor Rivera

**Affiliations:** ^1^Instituto de Ciencias Nucleares, Universidad Nacional Autónoma de México, Circuito Exterior, Mexico City, Mexico; ^2^Centro de Ciencias de la Complejidad, Universidad Nacional Autónoma de México, Circuito Mario de la Cueva 20, Insurgentes Cuicuilco, Mexico City, Mexico; ^3^Doctorado en Ciencias Biomedicas, Universidad Nacional Autónoma de México, Circuito Escolar, Ciudad Universitaria, Mexico City, Mexico; ^4^Facultad de Medicina, Universidad Nacional Autónoma de México, Circuito Interior, Ciudad Universitaria, Mexico City, Mexico; ^5^Facultad de Ciencias, Universidad Nacional Autónoma de México, Circuito Exterior, Ciudad Universitaria, Mexico City, Mexico

**Keywords:** metabolic syndrome, obesity, cross-sectional population study, education, age, body mass index (BMI), fasting blood test analysis

## Abstract

Metabolic disorders, such as obesity, elevated blood pressure, dyslipidemias, insulin resistance, hyperglycemia, and hyperuricemia have all been identified as risk factors for an epidemic of important and widespread chronic-degenerative diseases, such as type 2 diabetes and cardiovascular disease, that constitute some of the world's most important public health challenges. Their increasing prevalence can be associated with an aging population and to lifestyles within an obesogenic environment. Taking educational level as a proxy for lifestyle, and using both logistic and linear regressions, we study the relation between a wide set of metabolic biomarkers, and educational level, body mass index (BMI), age, and sex as correlates, in a population of 1,073 students, academic and non-academic staff at Mexico's largest university (UNAM). Controlling for BMI and sex, we consider educational level and age as complementary measures—degree and duration—of exposure to metabolic insults. Analyzing the role of education across a wide spectrum of educational levels (from primary school to doctoral degree), we show that higher education correlates to significantly better metabolic health when compared to lower levels, and is associated with significantly less risk for waist circumference, systolic blood pressure, glucose, glycosylated hemoglobin, triglycerides, high density lipoprotein and metabolic syndrome (all *p* < 0.05); but not for diastolic blood pressure, basal insulin, uric acid, low density lipoprotein, and total cholesterol. We classify each biomarker, and corresponding metabolic disorder, by its associated set of statistically significant correlates. Differences among the sets of significant correlates indicate various aetiologies and the need for targeted population-specific interventions. Thus, variables strongly linked to educational level are candidates for lifestyle change interventions. Hence, public policy efforts should be focused on those metabolic biomarkers strongly linked to education, while adopting a different approach for those biomarkers not linked as they may be poor targets for educational campaigns.

## 1. Introduction

In the last few decades, there has been a significant increase in the incidence of metabolic disorders, including disturbed glucose metabolism, general and abdominal obesity, elevated blood pressure, dyslipidemias, insulin resistance, hyperglycemia, and hyperuricemia that are all risk factors for several serious diseases, such as type 2 Diabetes Mellitus (DM2), cardiovascular disease (CVD), and stroke (1–4). Although these risk factors have a genetic component (5, 6), it is generally accepted that their current elevated incidence in most developed countries is due to a greatly increased exposure to metabolic insults that has at least two major sources: an obesogenic environment that may facilitate overconsumption, poor nutrition, and sedentarism, i.e., “life-style” (behavioral) factors (7–10); and aging populations, wherein the gradual appearance of metabolic disorders can occur due to longer lifespans (11, 12). Thus, aging can be thought of as a measure of the duration of metabolic insults and lifestyle a measure of their degree.

Metabolic biomarkers, as well as anthropometric and physiological variables, such as body mass index (BMI) and blood pressure, should reflect not only intrinsic genetic and physiological dependencies but also, importantly, metabolic insults due to the interaction between an individual and their environment, as intermediated by nutrition, activity, stress, and a host of other factors, which may differ between the sexes (13, 14). Indeed, risk factors for chronic diseases are widely classified as either *modifiable* or *non-modifiable*, with the former having been proven to cluster, as they are influenced by common sources, thus providing new insights to better target demographic profiles and inform public policies (15, 16). The complexity of human physiology, however, is such that the impact of a particular environmental interaction may be quite heterogeneous across different metabolic subsystems.

In terms of intervention and prevention, unlike age or sex, it is to modifiable risk factors, associated with behavior and lifestyle, that we must look to in order to make changes that will generate a positive impact on health status (17–19). Unfortunately, behavior and lifestyle, are highly complex and multi-factorial. To analyze them, various correlated, single variable proxies, such as educational level or socio-economic status, have been used in many studies. Educational level may be the more appropriate, being objective, easy to quantify, and capturing multiple facets of the social determinants of health, including personal behavior, living and working conditions, economic and social opportunities and resources (20). There is ample evidence that higher educational level is generally correlated with better health outcomes (20–26). In most studies, however, the spectrum of educational level in the associated populations is somewhat restricted, with either few participants at one extreme or the other. Additionally, most studies that have focused on the relation between health status and education, have also restricted attention to only one, or a few, health measures or biomarkers, such as BMI. For example, relevant to the context of the present study, the relation between BMI and educational level has been investigated in multiple studies (27–29), where it has been established that, in general, BMI is inversely related to educational level. Various studies have concluded that a more modest level of education is associated with a poorer general state of health (22–24).

Even though distinct metabolic factors have been individually linked to many diseases and, in particular, to CVD and DM2, overall metabolic risk has principally been addressed in the context of metabolic syndrome (MS) (30), which has been shown to be a useful tool for identifying individuals at risk of atherosclerotic CVD and DM2 (31–33), although there remains some controversy about its global significance (7, 34, 35), and as to whether its contribution to risk is equal or larger than the sum of its parts (36). While there exist different operational definitions of MS (30, 35, 37–39), that have been updated over time (13), most recent ones comprise a subset of the following (37): BMI, waist circumference (WC), total cholesterol, high density lipoprotein (HDL), triglycerides, glycemia, and high blood pressure—with specific thresholds for defining abnormal values. As well as the risk factors directly associated with MS, other metabolic biomarkers have been found to be of interest (40), such as glycosylated hemoglobin (Hb A1c) (41), homeostatic model assessment of insulin resistance (HOMA-IR) (42, 43), basal insulin (44, 45), and uric acid (46).

In this paper, we examine the dependence of a wide set of metabolic risk factors and biomarkers on educational level, BMI, age, and sex, within a unique Mexican university-based population that exhibits a particularly wide range of educational levels and ages. The overall aim of the study is to understand and predict obesity, and metabolic diseases over time by accumulating and analyzing a highly multi-factorial, multi-scale data set covering genetic, physiological, anthropometric, social, epidemiological, and psychological variables. However, in this paper we will use only transverse data to determine risk profiles for the considered metabolic variables, including anthropometric, blood pressure, and fasting blood test parameters, with special emphasis on how educational level and age are linked to metabolic risk. In particular, we will consider the impact of education beyond the undergraduate level, noting that it leads to additional health benefits. We explore several variables considered to be modifiable factors for MS (WC, HDL, blood pressure, triglycerides, and glucose) for which behavioral life-style changes are considered as first-line treatment (47). Although educational level has been found as a predictor variable associated with the odds of meeting healthy life-style guidelines, both in adults and senior populations (48), it is not clear if it is equally predictive for any metabolic biomarker and associated disorder.

## 2. Methods

### 2.1. Selection and Description of Participants

The present work is based on a cross-sectional, community-based health study in a population of students, academics and supporting staff of the Universidad Nacional Autónoma de México (UNAM), which is Mexico's largest university. Although the present study is part of a larger prospective investigation, here we will analyze the data of the initial baseline stage, taken in 2014, which consisted of 1,073 participants who were voluntarily enrolled into the study. Each participant provided a written informed consent. Besides filling in a substantial questionnaire, each participant had various vital signs [systolic blood pressure (SBP), diastolic blood pressure (DBP)] and anthropometric measurements (weight, height, WC) taken by trained medical staff using standard procedures. Additionally, they underwent a laboratory fasting blood test.

#### 2.1.1. Ethical and Human Research Considerations

This study was carried out in accordance with the recommendations of the Ethics Committee of the Facultad de Medicina of the UNAM, which approved the procedures and protocols for this study under project FM/DI/023/2014. All subjects gave written informed consent in accordance with the Declaration of Helsinki.

#### 2.1.2. Database

Study data is available in the UNAM repository at the web page: http://www.c3.unam.mx/health/, in either csv or database formats, and includes:

- *Demographic information*: sex, age, occupation (student, academic, or supporting staff), educational level, marital status, number of children, and siblings.
- *Anthropometric measures*: height, weight, and WC.
- *Blood pressure*: single (one-time) measurements of SBP and DBP.
- *Metabolic factors* determined by laboratory results of a fasting blood test: glucose, glycosylated hemoglobin (Hb A1c), basal insulin, total cholesterol, high-density lipoprotein (HDL), low-density lipoprotein (LDL), triglycerides, and uric acid levels.

From the original data set, 14 particular values associated with distinct variables were excluded, based on two main criteria:

1. outliers based on physiologically improbable or impossibly low values. This included removing from further analysis two glucose measurements, five values of HbA1c, and one each of insulin, HOMA, triglycerides, and uric acid;
2. values of HDL or LDL which were inconsistent with a given total cholesterol value. Three such values of LDL were removed from further analysis.

#### 2.1.3. Study Groups

The population was divided into five groups based on their “educational level,” as seen in Table 1:

1. *Primary* or elementary education, at most 6 years of school attendance (which in Mexico corresponds to the level called “primaria”).
2. *Secondary* or high-school, between 6 and 12 years of school attendance (which in Mexico includes “secundaria” and “preparatoria” levels).
3. *Undergraduate*, from 2 to 5 years of study after secondary level (which in Mexico includes “licenciatura” and “carrera técnica”).
4. *Master*, from 1 to 3 years of study after undergraduate level (which in Mexico comprises “maestría” and “especialidad”).
5. *Doctorate*, corresponds to the doctorate and postdoctoral levels, more than 4 years of study after master's level (which in Mexico are “doctorado” (Ph.D.) and “postdoc” levels).

**Table 1:** General description of the demographic data, anthropometric and blood pressure measurements of the UNAM study population.

**Descriptor**	**1**	**2**	**3**	**4**	**5**	**Total**
***n***	48	285	405	181	154	1,073
	4.5%	26.6%	37.6%	16.9%	14.4%	100%
Women *n*	35	190	264	116	84	689
(%)	(73%)	(67%)	(65%)	(64%)	(54%)	(64%)
Men *n*	13	95	141	65	70	384
(%)	(27%)	(33%)	(35%)	(36%)	(46%)	(36%)
**Age**	50 ± 13	43 ± 13	41 ± 12	42 ± 12	49 ± 13	43 ± 13
(years)	(24–77)	(19–74)	(20–80)	(24–67)	(29–81)	(19–81)
Women age	51 ± 13	43 ± 12	41 ± 12	42 ± 11	47 ± 13	43 ± 12
(years)	(24–77)	(20–74)	(20–80)	(24–67)	(29–81)	(20–81)
Men age	45 ± 14	42 ± 14	40 ± 12	41 ± 12	51 ± 12	43 ± 13
(years)	(25–62)	(19–72)	(21–74)	(27–67)	(29–76)	(19–76)
**Weight**	70 ± 14	71 ± 14	68 ± 15	66 ± 12	69 ± 14	69 ± 14
(kg)	(41–120)	(38–149)	(35–142)	(45–106)	(41–108)	(35–149)
Women weight	67 ± 11	67 ± 13	64 ± 13	62 ± 11	61 ± 10	64 ± 12
(kg)	(41–90)	(38–112)	(35–121)	(45–100)	(41–99)	(35–121)
Men weight	79 ± 17	78 ± 15	77 ± 16	74 ± 12	79 ± 10	77 ± 14
(kg)	(60–120)	(52–149)	(47–142)	(56–106)	(57–108)	(47–149)
**Height**	1.6 ± 0.1	1.59 ± 0.09	1.61 ± 0.09	1.62 ± 0.09	1.65 ± 0.09	1.61 ± 0.09
(m)	(1.41–1.90)	(1.39–1.96)	(1.24–1.97)	(1.42–1.89)	(1.44–1.90)	(1.24–1.97)
Women height	1.52 ± 0.06	1.55 ± 0.07	1.57 ± 0.07	1.58 ± 0.07	1.58 ± 0.06	1.56 ± 0.07
(m)	(1.41–1.65)	(1.39–1.90)	(1.42–1.97)	(1.42–1.89)	(1.44–1.70)	(1.39–1.97)
Men height	1.67 ± 0.09	1.68 ± 0.07	1.69 ± 0.08	1.71 ± 0.06	1.72 ± 0.06	1.69 ± 0.07
(m)	(1.54–1.90)	(1.48–1.96)	(1.24–1.90)	(1.60–1.84)	(1.60–1.90)	(1.24–1.96)
**BMI**	29 ± 4	28 ± 5	26 ± 5	25 ± 4	25 ± 4	26 ± 5
(kg/m^2^)	(17.1–39.6)	(15.6–51.8)	(13.3–50.6)	(15.1–38.5)	(16.5–35.9)	(13.3–51.8)
Women BMI	29 ± 4	28 ± 6	26 ± 5	25 ± 4	24 ± 4	26 ± 5
(kg/m^2^)	(17.1–37.5)	(15.6–51.8)	(13.3–46.7)	(15.1–38.5)	(16.5–35.9)	(13.3–51.8)
Men BMI	28 ± 4	28 ± 5	27 ± 6	25 ± 4	27 ± 3	27 ± 5
(kg/m^2^)	(22.3–39.6)	(18.3–42.6)	(15.2–50.6)	(18.2–38.0)	(21.4–34.9)	(15.1–50.6)
**WC**	96 ± 9	94 ± 12	92 ± 13	89 ± 12	92 ± 12	92 ± 12
(cm)	(71–116)	(63–134)	(60–153)	(64–162)	(64–167)	(60–167)
Women WC	95 ± 10	93 ± 12	90 ± 13	87 ± 13	87 ± 10	90 ± 12
(cm)	(71–115)	(63–134)	(60–149)	(64–162)	(64–112)	(60–162)
Men WC	97 ± 8	97 ± 11	95 ± 13	91 ± 11	98 ± 12	96 ± 12
(cm)	(85–116)	(73–128)	(70–153)	(66–119)	(78–167)	(66–167)
**SBP**	117 ± 17	115 ± 15	111 ± 15	112 ± 15	112 ± 13	113 ± 15
(mmHg)	(90–160)	(80–180)	(70–180)	(80–180)	(90–160)	(70–180)
**DBP**	76 ± 13	75 ± 11	73 ± 11	73 ± 11	74 ± 10	74 ± 11
(mmHg)	(60–100)	(50–110)	(50–110)	(50–110)	(60–90)	(50–110)
**Systemic PP**	41 ± 10	40 ± 10	38 ± 10	39 ± 9	39 ± 9	38 ± 10
(mmHg)	(20–70)	(20–100)	(10–90)	(20–70)	(10–90)	(10–100)

*Reported values for each group are mean ± standard deviation, and ranges (minimum–maximum)*.

*Participants are grouped by educational level: 1, primary; 2, secondary; 3, undergraduate; 4, master; 5, doctorate*.

### 2.2. Laboratory Procedures

Blood samples were obtained by trained medical professionals from participants who had fasted for at least 8 h and at most 12 h. Samples were stored at 4–5°C, and submitted for chemical analysis to obtain glucose, Hb A1c, basal insulin, total cholesterol, HDL, LDL, triglycerides, and uric acid levels. The analysis was performed at the Instituto Nacional de Ciencias Médicas y Nutrición “Salvador Zubirán.”

Fasting plasma glucose was measured using spectrophotometry and potentiometry with a hexokinase kit (amorting PIPES, NAD, Hexokinase, ATP, Mg^2+^, G6P-DH; AU 2700 Beckman Coulter®). Hb A1c was measured with High Performance Liquid Chromatography (HPLC) analysis with the Variant® Turbo kit 2.0, which consisted of 2 buffers and 1 wash solution. Fasting plasma insulin concentrations were determined using Chemiluminescence (Access Ultrasensitive Insulin, Unicell Dxl 800 Beckman Coulter®, Sensitivity: 0.03–300 U/mL). The lipid profile was obtained with enzymatic colorimetric assay (glycerol phosphate oxidase, cholesterol oxidase, accelerator-selective, detergent, and liquid-selective detergent). Uric acid was measured using the colorimetric method with uricase enzymatic OSR6698, system AU2700/5400, Beckmann Coulter®.

### 2.3. Derived Variables

Database information was used to calculate several derived parameters that are standard in clinical practice (49–51), such as BMI (52):

(1) $$\begin{array}{l} \text{BMI}=\frac{\text{weight}\left[kg\right]}{{\left(\text{height}\left[m\right]\right)}^{2}}, \end{array}$$

systemic pulse pressure (PP) (53):

(2) $$\begin{array}{l} \text{PP}=\text{SBP}-\text{DBP}, \end{array}$$

and the homeostasis model assessment insulin resistance (HOMA-IR) index (43, 54):

(3) $$\begin{array}{l} \text{HOMA}-\text{IR}=\frac{\text{basal insulin}\left[units/ml\right]\times \text{fast glucose}\left[mg/dL\right]}{18\times 22.5\left[mg/mldL\right]}. \end{array}$$

### 2.4. Healthy Parameter Ranges

For the anthropometric and biomarker measurements that are involved in the definition of MS by the International Diabetes Federation we used the cutoffs from Alberti et al. (55). For WC, we employed the suggested cutoffs for Ethnic South and Central Americans suggested in the same definition of MS (55) which have been used in other studies on Mexican populations (56). For PP, BMI, HOMA-IR, basal insulin, total cholesterol, LDL, Hb A1c, and uric acid we used cutoffs taken from the literature. These healthy norm value ranges are given in Table S1, where we also show the percentage of the studied population that exhibits abnormal values. MS was defined using the International Diabetes Federation worldwide definition (55) where central obesity was obligatory plus any two of:

1. Raised triglycerides ≥ 150 mg/dl.
2. Reduced HDL cholesterol < 40 mg/dl in men and < 50 mg/dl in women.
3. Raised blood pressure—SBP ≥ 130 mmHg or DBP ≥ 85 mmHg.
4. Raised fasting plasma glucose ≥ 100 mg/dl.

### 2.5. Statistics

The statistical analysis used in this study consisted first of simple population level diagnostics—means and standard deviations—as summary statistics of different subgroups. For analyzing the relation between the metabolic biomarkers and the correlates—educational level, age, BMI, and sex—we used both logistic and linear regressions (57, 58). Each measured biomarker was taken individually as the dependent variable. Logistic and linear regressions were performed for each one in relation to the independent variables: “educational level,” “BMI,” “sex,” and “age.” In the case of WC, BMI was not included as an independent variable due to its very high degree of collinearity with WC. Regression coefficients and their corresponding *p*-values were calculated, where we take the standard *p* < 0.05 as indicating that the relation does not support the null hypothesis that the independent and dependent variables are unrelated. However, given the recent debate and later position statement by the American Statistical Association (59) on statistical results based on *p*-values alone, we will not make definitive statements in our conclusions based only on such values. The class variable for the logistic regressions is the class of abnormal values associated with the thresholds seen in Table S1 which have been taken from the literature cited in the table. For example, for glucose, values above 100 mg/dL indicate the non-healthy class. For the linear regressions, in the case of age, due to observed non-linearities in the relations we also included a quadratic term and its corresponding regression coefficient to determine if this offered a better fit to the data. We also checked the variance inflation factors between educational level, age, and sex to check any degree of collinearity. All factors were very close to one.

We included both types of regressions as they offer complementary perspectives of the relations between the dependent and independent variables and, as such, significance in one does not necessarily imply significance in the other. The principal purpose of the logistic regressions is to determine those factors that are linked to metabolic disorders by calculating the odds ratios of the variables, whereas the linear regression is to determine to what extent the average level of a given metabolic factor is a function of age, BMI, and educational level, without emphasizing any relation to any definition of metabolic disorder.

## 3. Results

A general description of the study population is given in Table 1 (demographic, anthropometric measures, and blood pressure), and Table 2 (laboratory fasting blood analysis), where the population is divided into five groups according to their educational level (as described in section 2.1). Reported results include anthropometric measures, single (one-time) measurements of blood pressure, and the laboratory chemical analysis of the fasting blood test.

**Table 2:** General description of the laboratory fasting blood analysis of the UNAM study population.

**Descriptor**	**1**	**2**	**3**	**4**	**5**	**Total**
**Glucose**	114 ± 61	98 ± 36	97 ± 33	91 ± 14	98 ± 35	97 ± 34
(mg/dL)	(69–357)	(64–418)	(66–359)	(64–167)	(70–325)	(64–418)
**Hb A1c**	6 ± 2	6 ± 1	5 ± 1	5.1 ± 0.6	5 ± 1	5 ± 1
(%)	(4.4–15.0)	(4.0–14.4)	(3.7–15.2)	(4.0–9.8)	(4.0–12.8)	(3.7–15.2)
**Basal insulin**	10 ± 4	10 ± 8	8 ± 6	7 ± 5	7 ± 6	8 ± 6
(units/mL)	(2.8–21.8)	(1.7–63.2)	(1.1–55.8)	(0.9–33.3)	(1.4–45.2)	(0.9–63.2)
**HOMA-IR**	3 ± 1	3 ± 3	2 ± 2	2 ± 1	2 ± 2	2 ± 2
	(0.5–7.7)	(0.3–23.2)	(0.2–28.5)	(0.2–10.8)	(0.3–10.3)	(0.2–28.5)
**Uric acid**	6 ± 2	5 ± 1	5 ± 1	5 ± 1	5 ± 1	5 ± 1
(mg/dL)	(2.4–10.5)	(2.4–9.7)	(1.6–9.7)	(2.4–10.8)	(2.7–9.1)	(1.6–10.8)
Women	5 ± 1	5 ± 1	5 ± 1	5 ± 1	5 ± 1	5 ± 1
	(2.4–7.5)	(2.4–7.5)	(1.6–8.4)	(2.4–8.0)	(2.7–8.3)	(1.6–8.4)
Men	7 ± 1	7 ± 1	6 ± 1	6 ± 1	6 ± 1	6 ± 1
	(5.4–10.5)	(4.2–9.7)	(2.7–9.7)	(3.3–10.8)	(3.0–9.1)	(2.7–10.8)
**Triglycerides**	190 ± 110	180 ± 130	160 ± 94	150 ± 91	155 ± 99	160 ± 110
(mg/dL)	(60–565)	(38–988)	(27–642)	(38–695)	(42–766)	(27–988)
Women	175 ± 110	170 ± 110	140 ± 80	140 ± 80	129 ± 68	150 ± 92
	(60–565)	(42–988)	(27–606)	(38–695)	(42–379)	(27–988)
Men	240 ± 100	210 ± 150	180 ± 110	170 ± 99	190 ± 120	190 ± 122
	(87–408)	(38–845)	(41–642)	(40–535)	(51–766)	(38–845)
**Cholesterol**	210 ± 45	200 ± 46	200 ± 40	200 ± 42	210 ± 38	200 ± 42
(mg/dL)	(65–300)	(81–527)	(61–389)	(114–328)	(101–333)	(61–527)
Women	200 ± 48	200 ± 48	200 ± 40	200 ± 42	200 ± 40	200 ± 43
	(65–300)	(81–527)	(61–354)	(114–328)	(101–307)	(61–527)
Men	220 ± 38	200 ± 42	200 ± 42	200 ± 40	210 ± 36	200 ± 41
	(148–268)	(100–285)	(107–389)	(126–328)	(147–333)	(100–389)
**HDL**	45 ± 10	45 ± 11	47 ± 12	49 ± 13	52 ± 14	48 ± 12
(mg/dL)	(27–71)	(24–93)	(27–91)	(26–98)	(27–94)	(24–98)
Women	47 ± 9	47 ± 11	51 ± 13	52 ± 13	57 ± 15	51 ± 13
	(27–65)	(26–93)	(28–91)	(26–98)	(34–94)	(26–98)
Men	40 ± 10	40 ± 9	41 ± 8	44 ± 10	45 ± 10	42 ± 9
	(32–71)	(24–70)	(27–65)	(28–77)	(27–74)	(24–77)
**LDL**	127 ± 34	117 ± 36	120 ± 34	122 ± 34	127 ± 32	120 ± 35
(mg/dL)	(60.2–198.8)	(3.0–279.4)	(6.2–259.8)	(16.6–233.8)	(11.4–220.8)	(3.0–279.4)
Women	126 ± 33	118 ± 37	120 ± 33	120 ± 36	124 ± 31	120 ± 34
	(60.2–198.8)	(11.0–279.4)	(6.2–259.8)	(16.6–233.8)	(68.6–220.8)	(6.2–279.4)
Men	132 ± 38	120 ± 36	118 ± 37	125 ± 30	130 ± 34	120 ± 35
	(69.0–178.0)	(3.0–198.4)	(11.8–217.4)	(48.2–211.2)	(11.4–207.0)	(3.0–217.4)

*Reported values for each group are mean ± standard deviation, and ranges (minimum–maximum)*.

*Participants are grouped by educational level: 1, primary; 2, secondary; 3, undergraduate; 4, master; 5, doctorate*.

**Figure 1:**
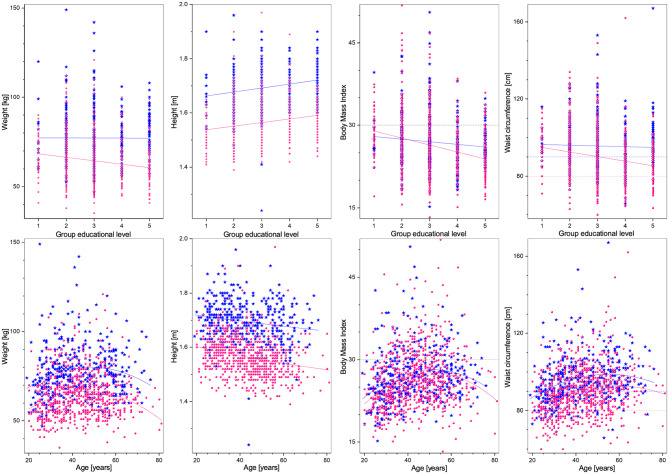
Anthropometric measures (from left to right: weight, height, BMI, and WC) as a function of educational level **(top)**, and age **(bottom)**. Distribution blue stars correspond to men, pink dots to women, continuous lines correspond to the best minimum least squares adjustment of the data, and dashed lines to cutoff values.

**Figure 2:**
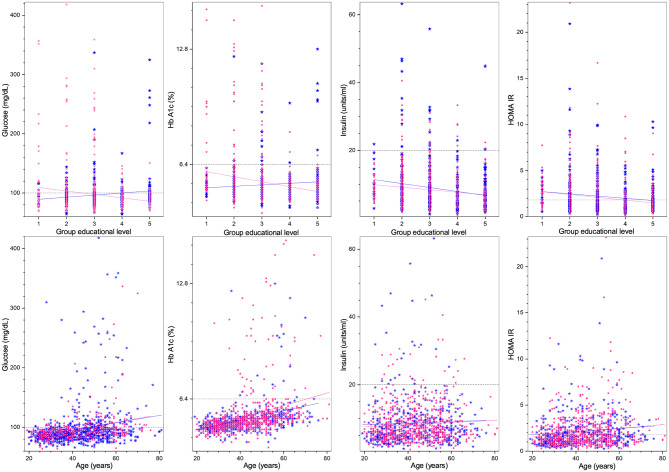
Laboratory fasting blood test associated with metabolism (from left to right: glucose, Hb A1c, insulin, HOMA-IR) as a function of educational level **(top)**, and age **(bottom)**. Distribution blue stars correspond to men, pink dots to women, continuous lines correspond to the best minimum least squares adjustment of the data, and dashed lines to cutoff values.

**Figure 3:**
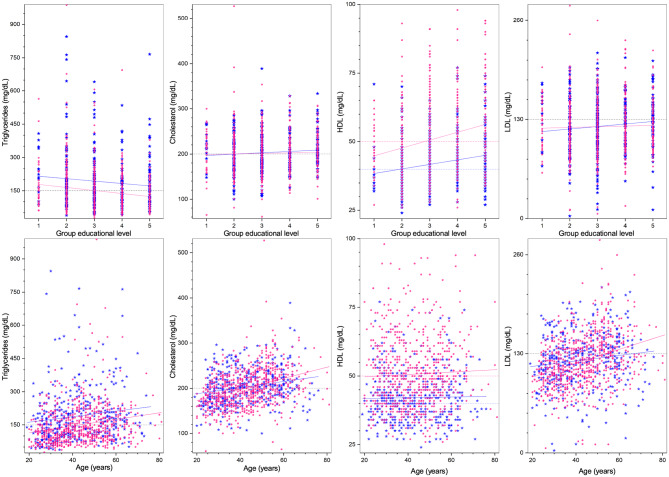
Laboratory fasting blood test associated with dyslipidemias (from left to right: total cholesterol, HDL, LDL, and triglycerides) as a function of educational level **(top)**, and age **(bottom)**. Distribution blue stars correspond to men, pink dots to women, continuous lines correspond to the best minimum least squares adjustment of the data, and dashed lines to cutoff values.

**Figure 4:**
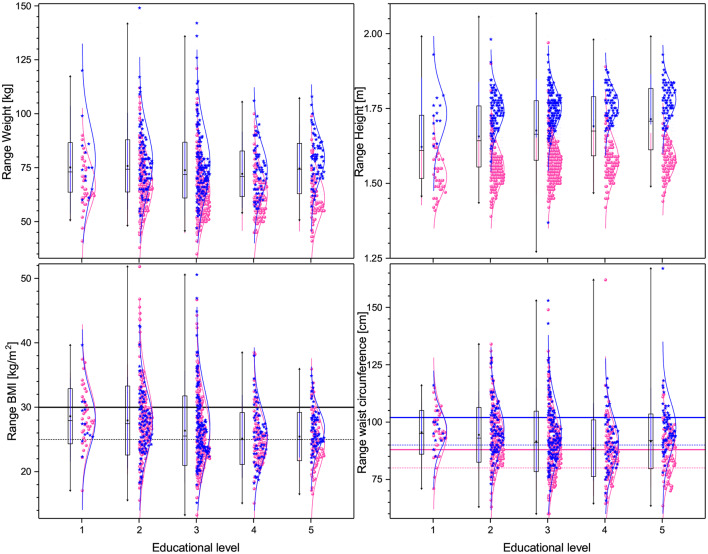
Box plots and distributions of the anthropometric measures for the different educational levels. **(Top)** panels show weight (left), height (right), while **(bottom)** panels show BMI (left) and waist circumference (right). Each box corresponds to one standard deviation around the mean (star). Distribution dots correspond to each subject, (pink for female and blue for male), the curve is the best Gaussian fit of the data, and horizontal lines correspond to thresholds defining health.

**Figure 5:**
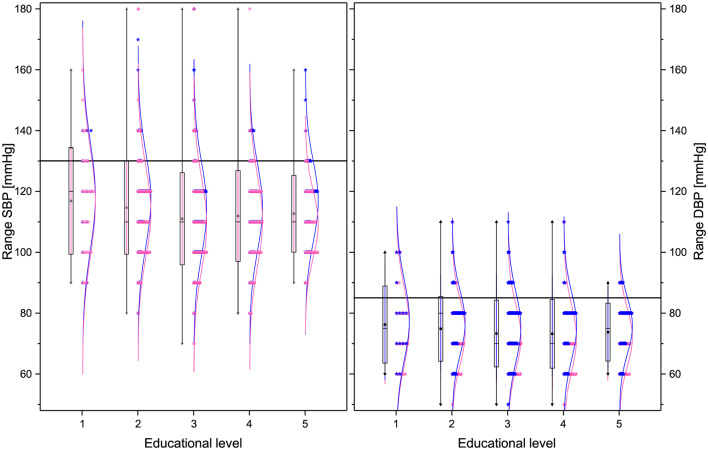
Box plots and distributions of the blood pressure parameters for the different educational levels. From left to right: SBP, and DBP. Each box corresponds to standard deviation around the mean (star). Distribution dots correspond to each subject (pink for female and blue for male), the curve is the best Gaussian fit of the data, and horizontal lines correspond to thresholds defining health.

**Figure 6:**
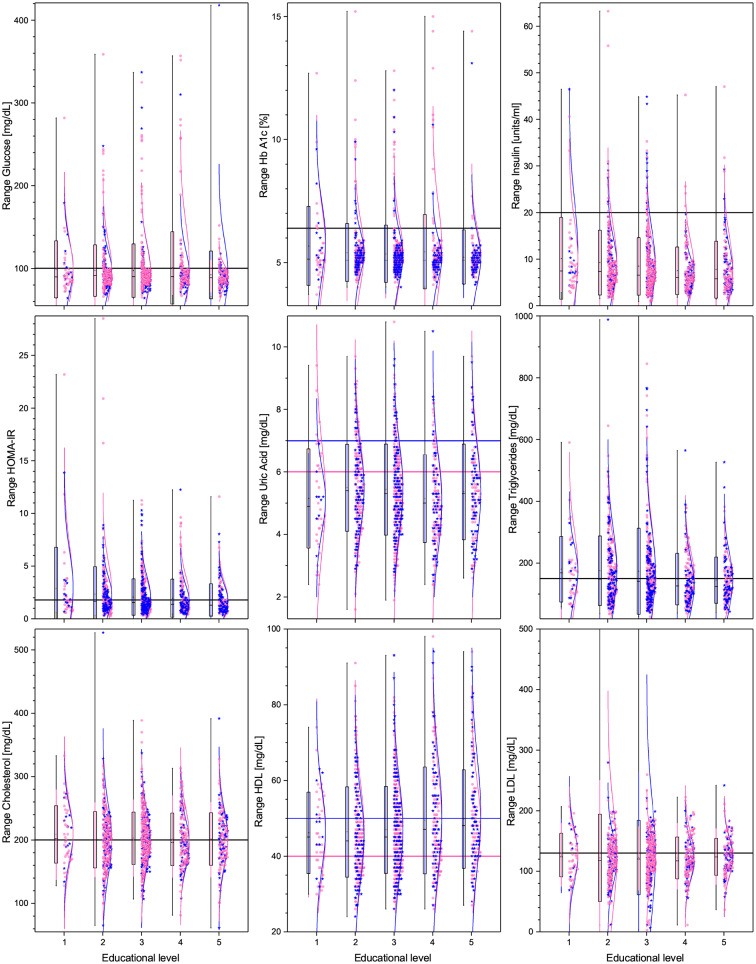
Box plots and distributions of the laboratory chemical analysis of the fasting blood test for the different educational levels. From top right to bottom left are: glucose, Hb A1c, insulin, total cholesterol, HDL, LDL, triglycerides, and uric acid levels. Each box corresponds to one standard deviation around the mean (star). Distribution dots correspond to each subject (pink for female and blue for male), the curve is the best Gaussian fit of the data, and horizontal lines correspond to thresholds defining health.

Regarding demographic characteristics, the split by sex is 64% women to 36% men, which is not uncommon in studies in Mexico (60, 61). As seen in Table 1, the highest (lowest) educational level groups have a higher (lower) proportion of men. With respect to the anthropometric measures (see Table 1, Figures 1, 4), we observe that those with higher educational level are generally taller, weigh less, have a smaller BMI, and smaller WC. The non-linear parabolic nature of the relation between weight, BMI, WC, and age is clearly visible in Figure 1, where we see that BMI, WC and weight tend to increase for older participants, with its highest value around 55 years old, while height decreases slightly. As a function of sex, females have a smaller WC, less weight, less BMI, and are shorter. Similar plots of the laboratory fasting blood test associated with metabolism (glucose, Hb A1c, basal insulin, and HOMA-IR) are seen in Figure 2 and associated with dyslipidemias (triglycerides, total cholesterol, LDL, and HDL) in Figure 3. Each plot shows the best fit regression for each relation. Further results using box-whisker plots, where the corresponding probability distributions are given as a function of educational level are shown in Supplementary Material for the anthropometric measures in Figure 4, blood pressure in Figure 5, and for the laboratory chemical analysis of the fasting blood test in Figure 6.

Results for the binomial regression taking educational level, BMI, age, and sex as independent variables are shown in Table 3, where we note that all abnormal class probabilities are decreasing functions of educational level and increasing functions of age, although in the case of WC for men the relation is not statistically significant at the 95% confidence level. In the case of blood pressure, Table 3 shows that the probability of abnormal values of SBP and DBP exhibits a strong dependence on educational level, with a decreasing odds ratio of 66% for every increase in educational level (*p* < 0.0005), while for DBP the corresponding decrease in odds ratio is 80% (*p* = 0.01). PP, on the other hand, is independent of educational level at the *p* < 0.05 level, but dependent on age (*p* = 0.007). As a function of age, we see that both SBP and DBP are affected, with odds ratio increases of 6.2 and 4.1% per year, respectively. Turning now to the metabolic parameters: we see that glucose (*p* = 0.013), Hb A1c (*p* = 0.003), HOMA-IR (*p* < 0.0005) all depend on educational level and on age (*p* < 0.0005 for all three factors), with odds ratios < 1 for educational level and >1 for age. Basal insulin depends neither on educational level (*p* = 0.23) nor on age (*p* = 0.975). For the lipid analysis, from Table 3 we see that triglycerides (*p* < 0.0005), and HDL for women (*p* < 0.0005) and for men (*p* < 0.0005), depend on educational level, whereas total cholesterol (*p* = 0.781) and LDL (*p* = 0.677) do not. On the contrary, although triglycerides (*p* < 0.0005) also depend on age, along with total cholesterol (*p* < 0.0005) and LDL (*p* < 0.0005), HDL for women (*p* = 0.190) and men (*p* = 0.383) do not. For uric acid, in Table 3 we see that it is independent of educational level for women (*p* = 0.285) and dependent for men (*p* = 0.032), and dependent on age for women (*p* = 0.024), and independent for men (*p* = 0.868).

**Table 3:** Binomial logistic regressions of anthropometric, blood pressure, and fasting blood test variables taking as class variable the at risk population using the cutoffs of Table S1, for the independent variables education (Edu), BMI, age, and sex.

**Variable**	***N***	**Edu**		**BMI**		**Age**		**Sex**	
		**exp(b)**	***p***	**exp(b)**	***p***	**exp(b)**	***p***	**exp(b)**	***p***
BMI	1,073	0.681	0.000^**^			1.021	0.001^*^	0.946	0.726
WC (Women)	689	0.700	0.000^**^			1.042	0.000^**^		
WC (Men)	384	0.912	0.384			1.047	0.000^**^		
SBP	1,073	0.760	0.022^*^	1.190	0.000^**^	1.065	0.000^**^	0.393	0.000^**^
DBP	1,073	0.939	0.491	1.167	0.000^**^	1.038	0.000^**^	0.614	0.016^*^
PP	1,073	0.848	0.507	1.129	0.004^*^	1.061	0.011^*^	0.382	0.087
Glucose	1,072	0.926	0.306	1.101	0.000^**^	1.055	0.000^**^	0.910	0.580
Hb A1c	1,068	0.767	0.034^*^	1.095	0.000^**^	1.064	0.000^**^	1.544	0.150
Insulin	1,072	1.068	0.679	1.203	0.000^**^	0.985	0.276	0.609	0.125
HOMA-IR	1,071	0.846	0.011^*^	1.240	0.000^**^	1.013	0.022^*^	1.075	0.624
Uric acid (Women)	689	1.025	0.832	1.106	0.000^**^	1.016	0.115		
Uric acid (Men)	383	0.856	0.140	1.110	0.000^**^	0.994	0.480		
Triglycerides	1,072	0.859	0.014^*^	1.091	0.000^**^	1.025	0.000^**^	0.469	0.000^**^
Total cholesterol	1,073	0.993	0.907	1.008	0.549	1.041	0.000^**^	0.880	0.335
HDL (Women)	689	0.770	0.001^*^	1.116	0.000^**^	0.983	0.012^*^		
HDL (Men)	384	0.737	0.002^*^	1.065	0.007^*^	1.002	0.797		
LDL	1,070	0.986	0.813	1.009	0.489	1.037	0.000^**^	0.805	0.109
Metabolic syndrome	1,073	0.827	0.005^*^	1.202	0.000^**^	1.036	0.000^**^	0.960	0.789

* *Indicates statistically significant at the p < 0.05 level*.

** *Indicates statistically significant at the p < 0.001 level*.

Finally, for MS, the odds ratio for educational level is 0.83 (*p* = 0.005), for BMI it is 1.20 (*p* < 0.0005), and for age 1.04 (*p* < 0.0005) but not on sex (*p* = 0.532). In Table S2, we show the incidence of MS in the different educational subgroups as well as the number and proportion in each group that have high WC but no other MS condition, using this as an indicator of better metabolic health. The importance of educational level is evident in the incidence of MS: 52% (Primary), 41% (Secondary), 32% (Undergraduate), 24% (Masters), and 26% (Doctorate) with an overall average of 33%. Taking the undergraduate level as reference, the difference between undergraduate and higher educational levels is significant, and the difference between undergraduate and lower levels is also significant (*p* = 0.005). Other risk measures follow a similar pattern. For example, for those at risk with a high WC, the percentages associated with presenting no other MS risk factor are: 16.3% (Primary), 17.0% (Secondary), 24.2% (Undergraduate), 29.6% (Masters), and 32.5% (Doctorate), where, again, the differences between undergraduate and higher levels are statistically significant (*p* = 0.006).

The results for the linear regressions are seen in Table 4. These results in general confirm the results determined using the logistic regression. For instance, we note that BMI and WC in women are very significant (*p* < 0.0005), whereas WC in men is not (*p* = 0.141), while the components of BMI—weight and height—are both significant, *p* < 0.0005 and *p* < 0.0005, respectively. As a function of sex, although, naturally, females have a smaller WC, less weight, and are shorter, their BMI for a given age is similar to that of men, with no statistically significant difference (*p* = 0.726). For blood pressure, Table 4 confirms that SBP depends on educational level (*p* = 0.001) and age (*p* < 0.0005), while for DBP the dependence on educational level is slightly weaker (*p* = 0.005). We also note there is a statistically significant difference between men and women for both SBP (*p* < 0.0005) and DBP (*p* = 0.005). Table 4 also confirms the dependence of glucose, Hb A1c, and HOMA-IR on educational level (*p* = 0.002, *p* < 0.0005, and *p* < 0.0005, respectively) and also on age (*p* < 0.0005 for all three factors). In this case, basal insulin is also dependent on educational level (*p* < 0.0005) and age (*p* = 0.033). Note that there are no significant differences between female and male subjects. Similarly, for lipids we see from Table 4, we see that triglycerides (*p* < 0.0005), HDL for women (*p* < 0.0005) and for men (*p* < 0.0005) are all dependent on educational level while total cholesterol (*p* = 0.357) and LDL (*p* = 0.261) are not. Similarly, age is significant for triglycerides (*p* < 0.0005), total cholesterol (*p* < 0.0005), and LDL (*p* < 0.0005) but not for HDL for women (*p* = 0.266) or for men (*p* = 0.738). Note that there is also a statistically significant difference between men and women for triglycerides (*p* < 0.0005). Finally, for uric acid, the linear regression yields a dependence on educational level for men (*p* = 0.002), but not for women (*p* = 0.170), and independence from age for both women (*p* = 0.254) and for men (*p* = 0.812).

**Table 4:** Multiple linear regressions of anthropometric, blood pressure, and fasting blood test variables using education (Edu), BMI, age, and sex as independent variables.

**Variable**	***N***	**Edu**	***p***	**BMI**	***p***	**Age**	***p***	**Sex**	***p***
Height	1,073	0.015	0.000^**^			−0.001	0.000^**^	−0.127	0.000^**^
Weight	1,073	−1.287	0.000^**^			0.077	0.014^*^	−13.127	0.000^**^
BMI	1,073	−1.03	0.000^**^			0.076	0.000^**^	−0.758	0.014^*^
WC (Women)	689	−2.389	0.000^**^			0.206	0.000^**^		
WC (Men)	384	−0.804	0.141			0.207	0.000^**^		
SBP	1,073	−0.331	0.383	1.009	0.000^**^	0.315	0.000^**^	−4.493	0.000^**^
DBP	1,073	−0.099	0.727	0.707	0.000^**^	0.164	0.000^**^	−3.047	0.000^**^
PP	1,073	−0.232	0.387	0.302	0.000^**^	0.150	0.000^**^	−1.446	0.015^*^
Glucose	1,072	−2.068	0.028^*^	0.733	0.000^**^	0.592	0.000^**^	−0.255	0.902
Hb A1c	1068	-0.137	0.000 ^**^	0.030	0.000 ^**^	0.030	0.000 ^**^	0.119	0.107
Insulin	1,072	−0.320	0.051	0.594	0.000^**^	−0.012	0.371	−0.175	0.628
HOMA-IR	1,071	−0.115	0.036^*^	0.177	0.000^**^	0.008	0.096	−0.029	0.813
Uric acid (Women)	689	0.039	0.317	0.073	0.000^**^	−0.001	0.668		
Uric acid (Men)	383	−0.128	0.028^*^	0.090	0.000^**^	−0.006	0.256		
Triglycerides	1,072	−9.909	0.001^*^	3.827	0.000^**^	1.183	0.000^**^	−41.313	0.000^**^
Total cholesterol	1,073	1.061	0.363	−0.005	0.983	1.004	0.000^**^	−2.428	0.346
HDL (Women)	689	1.926	0.000^**^	−0.733	0.000^**^	0.097	0.011^*^		
HDL (men)	384	1.428	0.001 ^*^	-0.408	0.000 ^**^	0.018	0.613		
LDL	1,070	0.973	0.323	−0.114	0.594	0.677	0.000^**^	−1.520	0.483

* *Indicates statistically significant at the p < 0.05 level*.

** *Indicates statistically significant at the p < 0.001 level*.

As a means of comparing the very similar results between the two types of regressions in Table 5 we show the relation between each metabolic biomarker and its corresponding set of statistically significant correlates, from among educational level (E), BMI (B), age (A), and sex (S), where for each metabolic factor, where a risk factor appears if it is significantly related at the *p* < 0.05 level. The asterisk denotes that the corresponding factor exhibits the same relation (increasing/decreasing risk) but is not significant at the *p* < 0.05 level with the sample size associated with the present study. Boldface denotes those variables involved in the definition of MS.

**Table 5:** Significant correlates for each metabolic biomarker from: Education (E), BMI (B), Age (A), and Sex (S), as determined from the odds ratios of the logistic regressions using the thresholds of Table S1, and multiple linear regressions.

**Metabolic biomarker**	**Significant correlates**	**Significant correlates**
	**logistic**	**linear**
**WC–women**	EA	EA
**WC–men**	E^*^A	E^*^A
**SBP**	EBAS	E^*^BAS
DBP	BAS	BAS
**Glucose**	E^*^BA	EBA
Hb A1c	EBA	EBA
Insulin	B	B
HOMA-IR	EBA	EBA^*^
Uric acid–women	B	B
Uric acid–men	E^*^B	EB
**Triglycerides**	EBAS	EBAS
Total cholesterol	A	A
**HDL–women**	EBA	EBA
**HDL–men**	EB	EB
LDL	A	A

* *Denotes that the corresponding factor indicates the same relation but is not significant at the 95% confidence level with this sample size. Boldface indicates those variables that enter in the definition of MS*.

## 4. Discussion

The fact that the highest/lowest educational levels have a lower/higher proportion of women reflects the preponderance of male faculty members in the university and the fact that, in Mexico, women have less opportunities to obtain higher education (62). At the educational extremes, for those in the lowest and highest education groups, the average age is higher than in the intermediate groups. For the lowest educational level - primary education only—this reflects the improvement in access to secondary education and beyond in Mexico in the last couple of decades, while the higher average age for postgraduates reflects the fact that the minimum age to obtain a PhD in Mexico is more than 27 years old. At the same time, the population considered is relatively homogeneous with respect to job-related activity levels, so that any substantial differences in activity are probably associated with voluntary activities.

The values of the metabolic variables we have considered are highly complex, multi-factorial functions, dependent on the interaction between an individual's physiology and their environment. Two important dimensions of this interaction are age and lifestyle, where the latter is itself complex and multi-factorial. We have here taken educational level to be a single proxy measure of lifestyle. Hence, we model the interaction between metabolism and environment using educational level and age. Additionally, we include BMI and sex as two important potential confounders. As age is not a modifiable factor, it is to educational level we must look for insight into the possibility of reducing metabolic risk. As such, we must first determine which metabolic variables are subject to variation as a function of educational level.

We first note that metabolic risk, as determined from the odds ratios of the logistic regressions, using the thresholds given in Table S1 (see Table 3), are increasing functions of BMI and age, and decreasing functions of educational level for all variables considered where conclusions can be made at the *p* < 0.05 significance level. We also see that the results of the linear regressions are in almost complete agreement with those of the logistic regressions (Table 5). Thus, within the confines of our results, we see that there is no metabolic variable where higher educational level correlates with an unhealthier metabolic state and, similarly, there is no variable where older age and higher BMI correlate with a healthier metabolic state. However, there is a great deal of heterogeneity as to which risk factors are significantly associated with a given metabolic variable, and as to the magnitude of the relation. Thus, the metabolic variables studied in this population have quite distinct risk profiles, as can be seen in Table 5. The principal profiles are EA, EB, EBAS, BAS, EBA, B, and A, where the appearance of a risk factor in the profile indicates a dependence of the corresponding metabolic variable on that risk factor, either with respect to a logistic or linear regression, where we emphasize again that independent/dependent here is with respect to the standard *p* < 0.05 and is therefore dependent on the sample size. The fact that the risk profiles are so distinct between the different components of MS has potential implications for understanding both its etiology and its characterization as a syndrome (34). Furthermore, their heterogeneity also implies that interventions need to be specifically tailored for each metabolic disorder.

Although many of the relations (metabolic variable—risk factor) that we study have been considered in the literature, a full understanding of the multi-factorial origin of the observed heterogeneity in the risk profiles for each metabolic factor requires much further study. Here we will restrict attention to some observations that we believe to be the most novel and relevant, beginning with the anthropometric variables. That BMI itself decreases significantly as a function of educational level is principally due to the fact that, in the case of men, although weight only decreases weakly, those of higher educational level are significantly taller. The relation between height and educational level is evidently multi-factorial. However, several potential relevant factors are: socio-economic status, wherein the higher the educational level, the more likely that person came from a family background of higher socio-economic status/educational level, and therefore a more nutritionally secure upbringing (20, 63, 64); and genetic factors, both specific to a Mexican population, where people at higher educational levels tend to have more European ancestry (65, 66). Thus, the fact that WC does not decrease with educational level in men, although BMI does, can be explained by the fact that the more educated men are significantly taller but only slightly heavier.

In the case of women, the increase in height, and the corresponding decrease in weight, as a function of educational level, provide an even greater effect on BMI when compared to men. The relation between education and BMI, especially in the case of women, has been observed in several studies (27, 29, 67). Our results are consistent with these findings. The relation between height and BMI vs. educational and socio-economic status has been considered in Tyrrell et al. (66), where it has been shown that the genetic component of height differences is relevant for the educational level and socio-economic status for men, but not for women where the genetic component of BMI is relevant for her socio-economic status. Taking as an example, social cultivation theory, where body standards are quite different for men and women, there is evidence that, for women, socio-economic status is inversely proportional to BMI in a much more significant way than for men, with height being more important for the latter (68).

Given the ample discussion in the literature as to the advantages and disadvantages of BMI vs. WC as a component of MS (69–71), we believe that this fact at least illuminates a potential defect of WC relative to BMI, especially in populations where there is a strong heterogeneity in height as a function of other variables, such as educational level. The principal reason is that WC depends not only on body shape, for example the distribution of visceral fat, but also on overall body size, whereas BMI and waist to height ratios are more attuned to being measures of body shape (Figure 4). Thus, not only are different thresholds relevant for WC due to genetic differences when comparing, say, an Asian with a northern European population, but also, as shown here, when considering populations with different educational levels. Additionally, using the recommended cutoffs for waist measurement for an Ethnic South or Central American population (55, 56), we note that 78.4% of the female population and 66.2% of the male population are above this threshold, while only 21.2% are above the threshold for BMI. This calls into question the use of this threshold for a Mexican population, as if such a large fraction of the population is defined as at risk with respect to this, or any other, factor, then it is not useful for stratifying risk between different populations.

Weight, BMI, and WC are increasing functions of age, and usually their risk analysis has involved studying only a linear relation, however, a regression that includes a quadratic term in age offers a statistically significant better fit than with only a linear term. Thus, weight, BMI, and WC are parabolic functions of age, increasing as a function of age (72) up to a maximum of 55–60 years old, and then decreasing. This parabolic shape (see Figure 1) is consistent with the fact that the chief risk group for adults is early adulthood, where most weight gain occurs (73).

The risk profiles of SBP and DBP are EBAS and BAS, respectively, showing that educational level is more relevant for SBP than DBP (see Figure 5, Table 5). The former depends on systolic debt (ventricular ejection volume), the surrounding vascular volume, and the distensibility of the arterial wall, whereas the latter only depends on the peripheral vascular resistance. Therefore, lifestyle habits, such as exercise and sodium consumption would be expected to affect more directly SBP (74). Indeed, a dependence on educational level has been noted in several studies in different countries, generally with the observation that higher levels of hypertension are associated with lower educational level (75–77). In the case of educational level, the unadjusted odds ratios for SBP and DBP, without controlling for BMI are 0.66 (*p* < 0.005) and 0.80 (*p* = 0.01), respectively. However, adjusting for BMI the corresponding odds ratios are 0.76 (*p* = 0.022) and 0.94 (*p* = 0.491) thus showing that a substantial fraction of the apparent dependence on educational level is due to changes in BMI. Other studies (78) have also shown a consistent relation between blood pressure and BMI, although the causal relations between high blood pressure and different anthropometric measurements is still poorly understood (79). We can also see that the age dependence is much stronger than the dependence on educational level, with standardized linear regression coefficients of 0.330 and 0.254 vs. −0.099 and −0.083 for SBP and DBP, respectively.

Turning to the lipid profiles: we note that both total cholesterol and LDL have profile A, depending on age but not on BMI or educational level (Table 5). In contrast, the profiles for HDL are EBA (women) and EB (men), from which we conclude that, in this population, HDL is much more sensitive to lifestyle, as proxied by education, than total cholesterol and LDL. The relative independence of these metabolic factors from educational level using the standard threshold has some worrying implications. Considering educational level as a potential proxy for “healthy” lifestyle, this implies that there is no evidence that lifestyles differ with respect to consumption, generation and elimination of LDLs, or of total cholesterol. Moreover, in distinction to other risk factors the percentage of the population at risk of dyslipidemias is already high, even in the younger adult population. For example, in the 19-29 age group the incidence of total cholesterol > 200 mg/dL is 29.3% and LDL > 130 mg/dL is 18.8%. Even more worryingly, the proportion of HDL < 50 (women) and < 40 (men) are 52.2 and 42.3%, respectively, for age group 19–29, which are the same as the averages over all ages. In the case of LDL and HDL, which are well known risk factors for atherosclerosis (31), this implies that the risk factors are potentially present at a relatively constant above-threshold levels over the entire life of a subset of individuals. Although longitudinal data would have to be acquired to test this hypothesis, it suggests that metabolic screening, even in younger adults, may be a cost-effective way of preventing MS and its consequences in later life.

It is well-known that HDL is found in larger concentrations in women than in men (80), in concordance with our results. This has been linked to lower levels of cardiovascular risk in women before menopause (81, 82), a difference which tends to level off with age (80, 83). However, the fact that, in the present study, we observe a statistically significant linear increase in HDL as a function of educational level for both men and women is interesting, as other studies have shown contradictory findings. For instance, in Benetou et al. (84), as part of the EPIC study carried out in Greece, it was shown that HDL levels decreased (increased) for men (women) as a function of educational level. Whereas, in another part of the EPIC study in the UK (85), lower levels of HDL were found in the least educated group for both men and women, with the effect being stronger for women. What is clear is that educational level as a single proxy variable for lifestyle, which is a highly multi-factorial construct, reflects different results for some metabolic risk factors due to the adaptive nature of the response of individuals of distinct educational levels to an evolving obesogenic environment which, in its turn, depends on an array of socio-demographic, socio-economic, and socio-cultural factors. Thus, for instance, the relationship between socio-economic status/educational level and cholesterol levels has led to conclusions that abnormal values are positively linked to these variables (86–88), inversely linked (84, 89, 90), or not linked at all (91) thus showing that the relationship is not rigidly universal but, rather, a reflection of the complexity of the multi-factorial interaction that the proxy variable is representing. This is further complicated by the fact that this relationship may change in time. For instance, as discussed in Benetou et al. (84), the relation between educational level and lipid levels changed over a period of about 20 years among the young Greek population studied, changing from a positive relationship between total cholesterol and educational level to a negative one.

Although we find no significant link between total cholesterol and educational level, there is a strong age dependence, which is due to several important age-dependent mechanisms associated with the metabolism of cholesterol. For example, it is known that as a person becomes older, there is a decrease in the hepatic hydroxylase cholesterol-alpha-1 responsible for the synthesis of bile acids, a diminishing of the LDL receptors, a decrease in the number of Niemann Pick C1 transporters that are responsible for mediating the intestinal absorption of cholesterol and its biosynthesis and, finally, the decrease of bacterial populations, which play a predominant role in the enterohepatic circulation of bile acids (92). Some studies have observed that total cholesterol tends to decrease in the last years of life, and that in the group with a total cholesterol below 3.0 mmol/L, mortality increases by 40–50% compared to subjects who have total cholesterol between 4.5 and 5.4 mmol/L (93).

Triglycerides in the blood come from three sources: ingestion in the diet, endogenous synthesis (hepatic lipogenesis), and release from fatty deposits (adipocyte turnover). The first source depends directly on behavior, and is responsible for more than 60% of the daily intake of lipids (94). while the latter are insulin regulated. As also described here, it has also been noted that the average concentration of triglycerides tends to be higher in men than in women, although there are many associated variables, such as apolipoprotein AV, which are associated with postprandial triglyceride levels (95). However, general food intake, alcohol consumption (96) and hyperadrenergic states (97) will modify triglyceride levels. The profile for triglyceride is EBAS, indicating that it is dependent on all four risk factors. These relationships between hypertriglyceridemia and educational level, age and sex have been observed in several studies (85), as has the relation between BMI and triglyceride levels (98). In this case, the impact of educational level is particularly important, with a regression coefficient of −9.909 compared to 3.827 for BMI, indicating that the reduction in triglyceride level from a unit increment in educational level is 2.59 times greater than the increment due to a unit increase in BMI, thus indicating the importance of interventions to modify the daily intake of lipids (94). Moreover, as with LDL and HDL, the incidence of hypertriglyceridemia in the youngest adults 19–29 years old is very high at 31.5%, and this is a significant risk factor for CVD due to the correlation of hypertriglyceridemia with coronary heart disease (99, 100).

For both glucose and Hb A1c the risk profile is EBA. The strong relation between BMI and glucose or Hb A1c has been noted in multiple studies. However, the dependence of fasting glucose level on educational level has been much less studied when compared to the relation with the incidence of DM2 (101). In one study, in a Korean population, incidence of fasting glucose levels > 100 mg/dL was found to be positively related to low educational level (102). Our results emphasize the importance of educational level as a protective factor as can be seen by comparing the regression coefficients for Hb A1c with respect to educational level and BMI, −0.137 and 0.030, respectively, indicating that a unit increase in educational level leads to a reduction in Hb A1c level that is 4.57 times larger than the corresponding increment due to a unit increase in BMI. The risk profile for basal insulin is B, indicating a dependence only on BMI. The fact that there is no significant dependence on age, neither in the case of the logistic regressions nor in linear regression, has been observed in other studies (103). In contrast to basal insulin, the risk profile of HOMA-IR is EBA, being a decreasing function of educational level and an increasing function of age and BMI, where a comparison of the regression functions and the odds ratios shows that BMI is the most important factor.

Uric acid comes from the final metabolism of purines, nitrogenous bases of DNA, and its concentration depends on both metabolism from food intake as well as its endogenous metabolism. The risk profiles for uric acid are EB for men and B for women. As noted here, it has been established that serum uric acid has higher average values in men than in women (104). Interestingly, uric acid increases in women but decreases in men as a function of educational level, though the statistical significance is weak in the case of women. Additionally, there is no significant dependence on age in either case but a statistically significant dependence on BMI (*p* < 0.001) as has been noted in other studies (105). The dependence of uric acid levels on age for women is potentially due to post-menopausal hormonal changes in uric acid levels (106). Increases in uric acid levels have been associated with insulin resistance (107), and as a risk factor for multiple diseases, such as DM2 (108–111), and MS (13, 112), as well as myocardial infarction, stroke, and congestive heart failure (113, 114).

Although we have taken educational level as a proxy for lifestyle, it is clearly not a direct causal factor. However, there are some obvious, and previously studied, variables that intermediate the relation between education and health, such as nutrition and exercise. Even if they have a direct causal connection with the metabolic factors we are considering, the relation between educational level and such lifestyle components is not clear. Do the better educated eat less? Eat healthier? Both? There is some evidence that the better educated both eat more healthily (115) and exercise more (116). Additionally, the relation between nutrition, exercise, and educational level will be affected by a host of socio-cultural factors, such that the relation between them is not necessarily generalizable across cultures.

There are many other factors that have been identified as variables that intermediate between education and health (117, 118). In particular, Mirowsky (119) concludes that “Education creates desirable outcomes because it trains individuals to acquire, evaluate and use information. It teaches individuals to tap the power of knowledge. As a result, education influences health in ways that are varied, present at all stages of adult life, cumulative, self-amplifying, and uniformly positive. Education develops the learned effectiveness that enables self-direction toward any and all values sought, including health.” We believe that this is fully in line with our results that education beyond the undergraduate level (effectively > 17 years of full time education) leads to very positive effects on metabolic health. This finding requires further research to identify what lifestyle characteristics, knowledge-base, or other factors, differentiate between subjects with an undergraduate and a postgraduate education. Obviously, one potentially important factor associated with our population is that it inhabits a very information rich environment—a university—but where there is a great deal of heterogeneity as to the degree of interaction with that information and, more importantly, how that information is incorporated into lifestyle and behavior. We would add some preliminary insights from our overall study that we believe to be relevant in potentially distinguishing between postgraduates and graduates: different eating habits, different degrees of health knowledge, more awareness of their physical/health state, greater disposition to change in the light of medical advice, more inclined to exercise, a more realistic perception of their health state, and they are taller—thus implying less excess consumption when compared to a smaller person. Some of these factors are physical in nature, but many point toward a difference in decision making.

As our results do not support the hypothesis that education is uniformly beneficial across all our considered metabolic variables, it must be determined why those most likely to live healthier lives fail in the case of certain variables, or is it that those variables are more difficult to improve, given a certain adjustment in lifestyle? If this is the case, public policy efforts should be focused on education-susceptible variables, prone to modification by exposure to information and intuitively incorporated into impactful life-style changes, while adopting a different approach for education-non-susceptible variables which are poor targets for educational campaigns. Although certain metabolic elements do not show significantly reduced risk for increasing educational level, overall metabolic health, as measured by the incidence of MS, is improved. Moreover, for those with one risk factor, the probability to have a second, or more risk factors, is significantly less. Thus, the more educated avoid a clustering of metabolic risk factors. Furthermore, as our population covers a large range of educational levels, we see that metabolic health keeps on improving beyond the undergraduate level, with masters/doctoral level participants being metabolically healthier than undergraduate level participants.

As well as a significant heterogeneity in metabolic risk profile as a function of educational level, we also see a significant heterogeneity in age, with the distribution of risk across the spectrum of factors for younger adults (19–29 years old) being quite different to that of older (> 40 years old) people. In particular, we saw that, in strong contrast to hyperglycemia, there were substantial fractions of young adults exhibiting dyslipidemias that were significant contributors to MS. This heterogeneity in age of metabolic risk implies that public health interventions also need to be specifically tailored to each age group.

## 5. Study Limitations

There are several limitations of the study. Firstly, as in the context of this paper, it is a cross-sectional study, we can make no inference as to any causal effects as a function of age. More specifically, the metabolic state of an individual is a result of their intrinsic physiology and their interaction with a changing environment over their lifetime. Although we do not know how that environment has changed, we can make the assumption that, for two individuals of the same age, the differences can be studied using educational level as a measure of the differences in the interaction. Additionally, even in a prospective study, it would be very difficult to track a set of environmental descriptors that proffered a sufficiently rich description of those aspects of the multi-factorial interaction between an individual and their environment that most affect metabolic state. Another limitation is that the population we consider is not representative of the wider Mexican population. However, we also believe that this is a strength. First of all, with respect to demographic characteristics it is highly heterogeneous, especially in terms of educational level, ranging from a primary only education up to postgraduate and therefore permits the explicit analysis of the impact of education beyond the undergraduate level as has been a chief objective of this paper. We may also observe differences due to educational level in a relatively uniform environment, where significant differences in the work place, such as in work-activity levels, or stress associated with job security, might be expected to be relatively small. Additionally, we believe that there are cross-cultural similarities between individuals at the higher levels of education that would indicate potentially similar results in analogous populations elsewhere. Another limitation is that educational level in this population is very much correlated with socio-economic status as measured by income. However, even if educational level was just a proxy for socio-economic status it would still be necessary to further investigate which specific lifestyle factors enabled postgraduates to be metabolically healthier than graduates. Finally, we are also assuming that educational level is a meaningful proxy for a large number of factors that constitute an individual's lifestyle.

## 6. Conclusion

In this paper we have shown that each metabolic biomarker and associated disorder has its own corresponding risk profile in terms of educational level, BMI, age, and sex and that these profiles can be used to classify metabolic disorders. Taking BMI as a control variable, educational level and age represent two complementary measures of exposure to metabolic insults, with age representing cumulative exposure and educational level, as a lifestyle proxy, representing the degree of exposure. Metabolic disorders can then be classified according to their dependence/independence on educational level and/or age taken as measures of degree and duration of metabolic insult exposure. We determined that in women WC, SBP, glucose, Hb A1c, HOMA-IR, triglycerides, and HDL depend on both degree and duration; whereas in men WC, DBP, total cholesterol, and LDL depend only on duration; uric acid and HDL only on degree, while in women basal insulin and uric acid depend on neither. Interestingly, all components of MS depend on both degree and duration except in the case of HDL.

The distinct risk profiles potentially indicate different aetiologies for the different disorders and this is also indicated by their quite different prevalence as a function of age. Moreover, we conclude that the distinct profiles indicate that both clinical and public health interventions for a given metabolic disorder need to be tailored to age and education (lifestyle) specific groups. The fact that educational level correlates with better health in many, but not all, of the metabolic variables and, in particular, in the components of MS, requires much more study to determine those characteristics that differentiate the lifestyle decisions of one educational group vs. another. In particular, given the better metabolic health of postgraduates vs. graduates, what are the differentiating factors and/or behaviors that lead to such a result?

## Data Availability Statement

The datasets for this study can be found in http://www.c3.unam.mx/health/.

## Ethics Statement

The studies involving human participants were reviewed and approved by Ethics Committee of the Facultad de Medicina of the Universidad Nacional Autónoma de México under project FM/DI/023/2014. The patients/participants provided their written informed consent to participate in this study.

## Author Contributions

All authors wrote, revised the manuscript, and approve its submission for publication, certifying that the work is original and their own. CS conceived and designed the investigation, questionnaires, and protocols. AR-C, AH-C, RM-T, AB-M, and LC performed the data acquisition. JL-R made database. JE, LC, JL-R, and AR analyzed the data. CS, JE, AR-C, AB-M, LC, RF, and AR results interpretation and discussion.

## Conflict of Interest

The authors declare that the research was conducted in the absence of any commercial or financial relationships that could be construed as a potential conflict of interest.

## Abbreviations

DM2: type 2 diabetes mellitus
CVD: cardiovascular disease
MS: metabolic syndrome
BMI: body mass index
WC: waist circumference
HDL: high density lipoprotein
LDL: low density lipoprotein
Hb A1c: glycosylated hemoglobin
HOMA-IR: homeostatic model assessment of insulin resistance
SBP: systolic blood pressure
DBP: diastolic blood pressure
PP: systemic pulse pressure.
