# Supplementary material for: The Impact of Education and Age on Metabolic Disorders
Source: Front Public Health. 2020 May 20;8:180. doi: 10.3389/fpubh.2020.00180 (PMC7326131; doi:10.3389/fpubh.2020.00180)
Supplement: Supplementary file 1 [file Data_Sheet_1.PDF]

Supplementary Material:
Metabolic risk and the role of higher education

1 SUPPLEMENTARY TABLES

Table S1. **Reference values for anthropometric measurements, blood pressure, and fasting chemical blood analysis.**

Variable	Normal level	Cutoff value	Units	% > cutoff	Reference
**BMI**	18.5 to 29.9	≥ 30	kg/m^2	21.1%	[1,2]
**WC**					
women	≤ 80	> 80	cm	78.4%	[3]
men	≤ 90	> 90	cm	66.3%	[3]
**SBP**	120 to 130	> 130	mmHg	7.3%	[1,4]
**DBP**	60 to 85	> 85	mmHg	12.6%	[1,4]
**PP**	30 to 60	> 60	mmHg	1.3%	[1,4]
**Glucose**	70 to 100	> 100	mg/dL	20.2%	[2]
**Hb A1c**	< 5.7	> 6.4	%	6.0%	[2,5]
**Basal insulin**	2 to 20	> 20	units/ml	4.3%	[6-8]
**HOMA-IR**	≤ 1.8	> 1.8		39.8%	[6,9]
**Uric acid**					
women	≤ 6	> 6	mg/dL	12.0%	[10,11]
men	≤ 7	> 7	mg/dL	31.5%	[10,11]
**Triglycerides**	40 to 150	> 150	mg/dL	44.3%	[1]
**Total cholesterol**	140 to 200	> 200	mg/dL	48.9%	[12]
**HDL**					
women	≥ 50	< 50	mg/dL	52.2%	[12]
men	≥ 40	< 40	mg/dL	42.3%	[12]
**LDL**	≤ 130	> 130	mg/dL	37.6%	[1]

In Table S2 below we show the incidence of MS in the different educational subgroups. Taking as reference level Primary, all higher levels are statistically significantly ($p < 0.05$) lower in MS incidence than the Primary group. Indeed, each higher group has significantly less incidence of MS than any lower group below it except in the case of Undergraduate versus Secondary where the difference does not reach the 95% confidence level and with Masters versus Doctoral level, where the difference is attributable to the substantially higher average age in the doctoral group versus the Masters group, as can be seen in Table 1. In this case, age is a confounding variable. The binomial regression controls for this confounding factor and there we may conclude that controlling for age the Doctoral level has a lower incidence of MS than the Masters level.

Similarly, we show that for those at risk with a high WC, the percentages associated with presenting no other MS risk factor. We may observe that the percentages with better metabolic health increase as a function of educational level with the differences again being statistically significant relative to the Primary group as reference.

Table S2. Incidence of Metabolic Syndrome for the different educational subgroups

Educational level	1	2	3	4	5	Total
Number with MS	25	108	138	36	39	346
% with MS	52.1%	37.9%	34.1%	19.9%	25.3%	32.2%
Number WC only	7	39	30	34	38	148
% WC only	16.3%	17.0%	24.2%	29.6%	32.5%	18.6%

Participants are grouped by educational level: 1-primary, 2-secondary, 3-undergraduate, 4-master, 5-doctorate.

2 SUPPLEMENTARY FIGURES

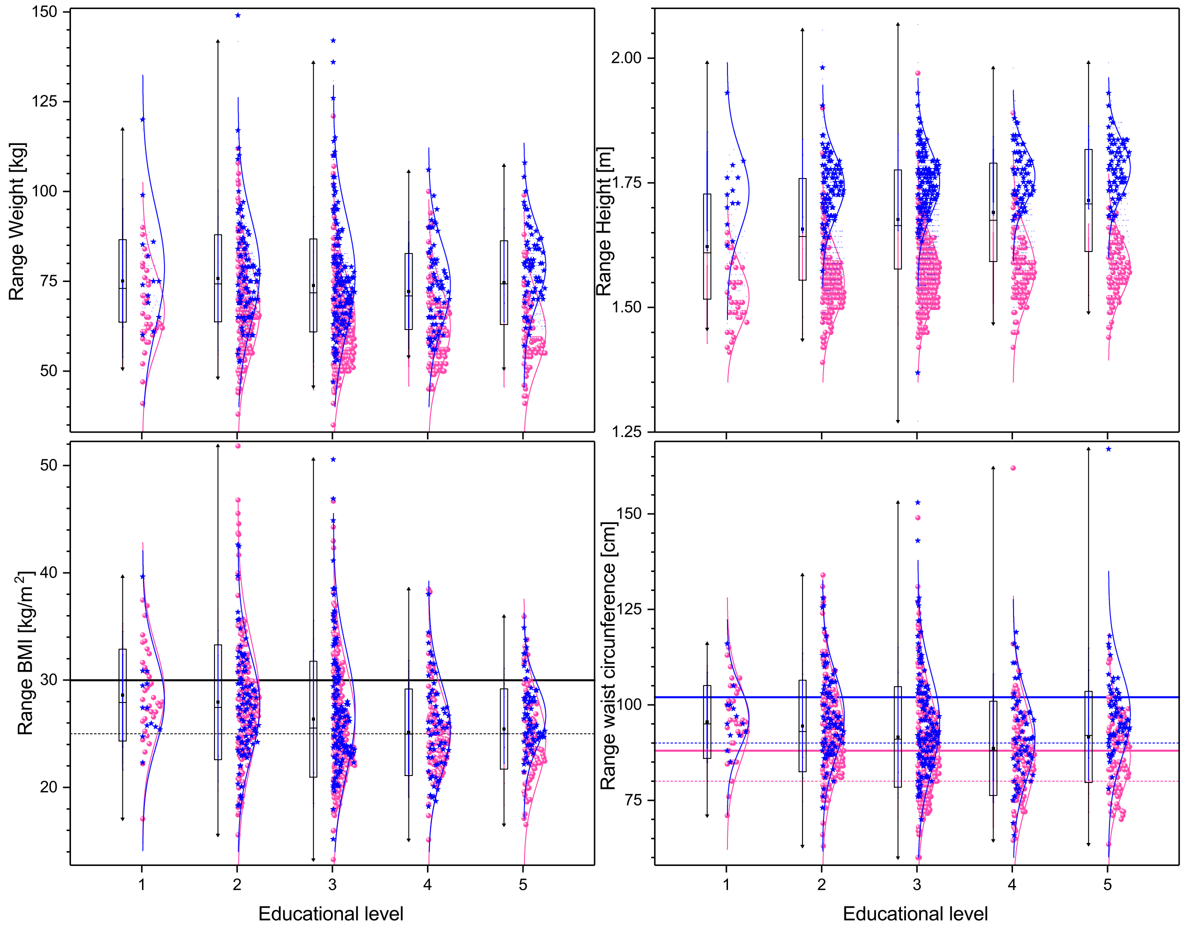

Figure S1. Box plots and distributions of the anthropometric measures for the different educational levels. Upper panels show weight (left), height (right), while bottom panels show BMI (left) and waist circumference (right). Each box corresponds to one standard deviation around the mean (star). Distribution dots correspond to each subject, (pink for female and blue for male), the curve is the best Gaussian fit of the data, and horizontal lines correspond to thresholds defining health.

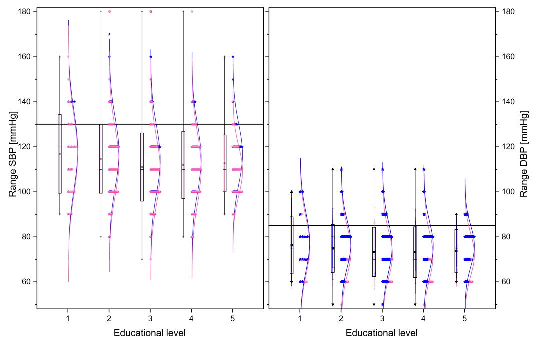

Figure S2. Box plots and distributions of the blood pressure parameters for the different educational levels. From left to right: SBP, and DBP. Each box corresponds to standard deviation around the mean (star). Distribution dots correspond to each subject (pink for female and blue for male), the curve is the best Gaussian fit of the data, and horizontal lines correspond to thresholds defining health.

REFERENCES

1. K.G.M. Alberti, P. Zimmet, J. Shaw. (2006). Metabolic syndrome–a new world–wide definition. A consensus statement from the International Diabetes Federation, *Diabetic Medicine*, **23** (5): 469–480.

2. American Diabetes Association. (2014). Diagnosis and classification of diabetes mellitus, *Diabetes Care*, **37** (S1): S81–S90.

3. World Health Organization. (2008). Waist circumference and waist–hip ratio: report of a WHO expert consultation, Geneva, 8-11 December 2008.

4. A.S. Fauci, et al. Harrison's principles of internal medicine, (Mcgraw-hill, New York, 2008.)

5. American Diabetes Association. (2015). Glycemic targets. Sec. 6. In Standards of Medical Care in Diabetes, *Diabetes Care*, **38** (S1): S33–S40.

6. M. Stumvoll, A. Mitrakou, W. Pimenta, T. Jenssen, H. Yki-Järvinen, T. Van Haeften, et al. (2000). Use of the oral glucose tolerance test to assess insulin release and insulin sensitivity, *Diabetes Care*, **23** (3): 295–301.

7. J. Pettus, T. Santos Cavaiola, W.V. Tamborlane, S. Edelman. (2016). The past, present, and future of basal insulins, *Diabetes/metabolism Research and Reviews*, **32** (6): 478–496.

8. J.D. Bagdade, E.L. Bierman, D. Porte. (1967). The significance of basal insulin levels in the evaluation of the insulin response to glucose in diabetic and nondiabetic subjects, *Journal of Clinical Investigation*, **46** (10): 1549–1557.

9. T.M. Wallace, J.C. Levy, D.R. Matthews. (2004). Use and abuse of HOMA modeling, *Diabetes Care*, **27** (6): 1487–1495.

10. X. Sui, T.S. Church, R.A. Meriwether, F. Lobelo, S.N. Blair. (2008). Uric acid and the development of metabolic syndrome in women and men, *Metabolism*, **57** (6): 845–852.

11. M.F. Amaya-Villalva, G. González-Aguilar, O. Rouzaud-Sánchez, S. Gorinstein, H. Astiazarán-García, M. Robles-Sánchez, (2015). Obesity–related indicators and their relationship with serum antioxidant activity levels in Mexican adults, *Nutricion Hospitalaria*, **31** (5): 1989–1995.

12. K.G.M.M. Alberti, P. Zimmet, J. Shaw. (2005). The metabolic syndrome–a new worldwide definition, *Lancet*, **366** (9491): 1059–1062.

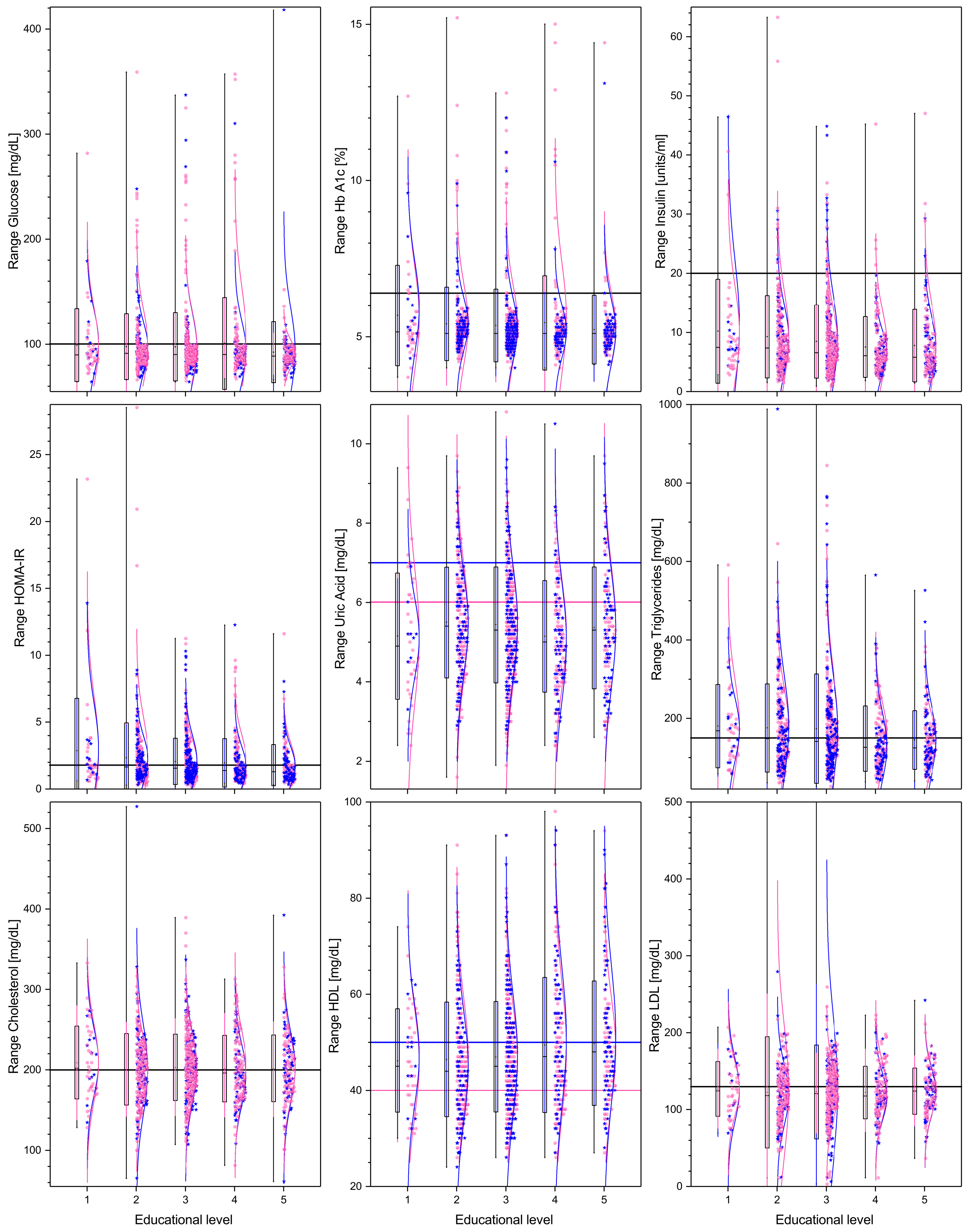

Figure S3. Box plots and distributions of the laboratory chemical analysis of the fasting blood test for the different educational levels. From top right to bottom left are: glucose, Hb A1c, insulin, total cholesterol, HDL, LDL, triglycerides, and uric acid levels. Each box corresponds to one standard deviation around the mean (star). Distribution dots correspond to each subject (pink for female and blue for male), the curve is the best Gaussian fit of the data, and horizontal lines correspond to thresholds defining health.
